# A Review on Innovative Biotechnological Approaches for the Upcycling of Citrus Fruit Waste to Obtain Value-Added Bioproducts^§^

**DOI:** 10.17113/ftb.63.02.25.8735

**Published:** 2025-06

**Authors:** Mahalingam Divyasakthi, Yerasala Charu Lekha Sarayu, Dilip Kumar Shanmugam, Guruviah Karthigadevi, Ramasamy Subbaiya, Natchimuthu Karmegam, J. Jessica Kaaviya, Woo Jin Chung, Soon Woong Chang, Balasubramani Ravindran, Kuan Shiong Khoo

**Affiliations:** 1 Department of Biotechnology, Sri Venkateswara College of Engineering, Pennalur Sriperumbudur (Tk), Kancheepuram District, Tamil Nadu 602117, India; 2 Department of Chemical Engineering, Indian Institute of Technology Madras, Chennai, India; 3 Department of Biological Sciences, School of Mathematics and Natural Sciences, The Copperbelt University, Riverside, Jambo Drive, P O Box, 21692, Kitwe, Zambia; 4 Post Graduate and Research Department of Botany, Government Arts College (Autonomous), Salem 636 007, Tamil Nadu, India; 5 Department of Biotechnology, Alagappa College of Technology, Anna University, Chennai India; 6 Department of Civil and Energy System Engineering, Kyonggi University, Suwon 16227, Korea; 7 Department of Microbiology, Faculty of Arts Science, Commerce and Management, Karpagam Academy of Higher Education, Coimbatore, Tamil Nadu, India.; 8 Department of Chemical Engineering and Materials Science, Yuan Ze University, Taoyuan, Taiwan

**Keywords:** citrus waste, sustainability, bioactive compounds, antioxidants, biorefinery, green extraction techniques, circular economy

## Abstract

The cultivation of citrus fruits has increased significantly around the globe due to rising consumer demand. The citrus fruit processing industry produces approx. 110 to 120 million tonnes of citrus fruit waste worldwide every year. This in turn contributes to landfills and environmental pollution, and poses a risk to human health and the ecosystem. Proper recycling of citrus waste helps reduce pollution and also serves as a sustainable source for the production of different bio-based products. Abundant bioactive compounds in citrus waste offer immense economic value for the production of various useful products. Moreover, bioactive compounds found in citrus wastes have various biological properties, including antioxidant, anticancer, antimutagenic, antiplatelet, cardioprotective and antiviral activities. Instead of disposing of them directly, citrus wastes can be upcycled into various value-added products, including single-cell proteins, biopolymers, pectin, biofuel, biofertilizer and bioenergy. Citrus peels serve as a cost-effective reservoir of nutraceuticals and provide an affordable dietary option for the treatment of degenerative diseases. The citrus waste, which is used as a biofertilizer and is a rich source of phenolic compounds and carotenoids, helps to extend the shelf life of food. The aim is to maintain economic viability and sustainability with the help of recent innovations in the industry. This review discusses recent advances in the valorization of citrus fruit waste and presents innovative biotechnological approaches to extract valuable bioactive compounds such as limonene, flavonoids and pectin. These compounds are used in different sectors, from the food and pharmaceutical industries to bioenergy. Techniques such as microwave-assisted extraction (MAE) and ultrasound-assisted extraction (UAE) are characterized by high yields and energy efficiency. Techniques for sampling, pretreatment, extraction of phytochemicals, purification and identification of citrus fruit waste are also studied. Additionally, this review highlights the environmental benefits of waste valorization as part of a circular economy approach that contributes to both economic sustainability and pollution reduction.

## INTRODUCTION

**Fig. 1:**
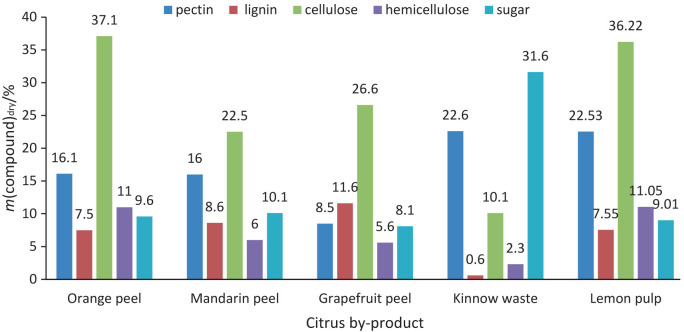
Proximate composition of byproducts of citrus fruits (*7*, *8*)

Citrus fruits, which belong to the *Rutaceae* family, are cultivated in all tropical and subtropical regions (*1*). Brazil, China and the United States are the largest producers of citrus fruits, followed by India, Mexico and Spain (*2*). Northern Myanmar and some southern parts of the Himalayas are considered to be the true origin of *Citrus* (*1*). Important *Citrus* species of this family are *Citrus sinensis* (sweet orange), *Citrus medica* (citron), *Citrus limon* (lemon), *Citrus aurantifolia* (lime), *Citrus paradisi* (grapefruit), *Citrus aurantium* (sour orange), *Citrus reticulata* (tangerine) and *Citrus maxima* (shaddock or pomelo) (*3*). Citrus fruits are rich in vitamin C and also contain a variety of beneficial vitamins and nutrients, including folate, thiamine, minerals, fibre, carotenoids, flavonoids and limonoids. There is substantial data that shows that consuming citrus fruits could reduce the risk of cardiovascular disease and obesity and contribute to mass reduction. In 2020, the annual production of citrus fruits was 124.4 million tonnes, with China being the largest producer, accounting for 28.66 % of the overall citrus yield (*4*). Although citrus fruits are mainly consumed raw for their health benefits, one-third of the toal production is processed to some extent (*5*). Peel, seeds, pulp and segment residues make up 50 to 60 % of the fruit composition and are recovered after processing the fruit. About 80 % of citrus peel contains moisture and is divided into mesocarp/albedo (a white soft layer) and flavedo/epicarp (the coloured outer surface). The albedo is the source of pectin, while the terpenoids are extracted from the flavedo. The polysaccharides in the cell wall of the peel consist of three main components: pectin, cellulose and hemicelluloses (*6*). Fig. 1 (*7*, *8*) shows the composition of citrus byproducts, including sugar, cellulose, hemicellulose, lignin and pectin, from different citrus species.

Different phytochemicals are formed during the growth phase of the fruit, which include relatively low-molecular-mass phenolic compounds, terpenoids, stilbenes, acetophenone, flavonoids and tannins (in condensed form) (*7*). The peels of *Citrus sinensis* are abundant in fibre, vitamin C, phenolic compounds and flavonoids, which serve as potent antioxidants. Nevertheless, these citrus residues are typically discarded as waste. The byproducts include seeds, pulp and peels and they are considered harmful for the environment, human health and aquatic life when not disposed properly because of their organic nature (*1*). Conventional methods for managing citrus peel waste include depositing it in landfills, utilising it for composting, extracting pectin and as animal feed. But some of these alternatives are not environmentally friendly and cost effective. Thus, there is a need to address this issue (*9*). Citrus is rich in bioactive compounds. Phenolic compounds in citrus peels act as antioxidants and prevent free radical damage to cells (*9*). Flavonoids and plant secondary metabolites have anticancer, anti-inflammatory and neuroprotective properties. Studies have shown that flavonoids are associated with a reduced risk of developing inflammatory bowel disease (IBD) and other degenerative disorders. Essential oils found in the citrus fruits are categorised mainly into oxygenated monoterpenes, oxygenated sesquiterpenes, hydrocarbon monoterpenes and hydrocarbon sesquiterpenes (*10*). With the growing popularity of bioactive components and the concept of functional food, foods enriched with citrus peel have come on the market (*11*). Citrus peel wastes are also exploited for the preparation of enzymes and bioflocculants. On the other hand, citrus seeds are made up of two main constituents: 14 % protein and 36 % seed oil (*12*). Citrus seeds have a significant nutritional value and oil content, making them valuable for use as food supplements or pharmaceuticals (*3*). Citrus waste is best suited as dietary supplements as these non-edible components are inexpensive and easily accessible (*13*). The fruit of *Citrus bergamia*, bergamot is exclusively used in the production of essential oil that is extracted from its peel. Due to its notable antibacterial and antiseptic properties, citrus finds extensive application in the pharmaceutical industry. Citrus waste is used as a fragrance in cosmetics, for example in soaps and perfumes. In the food industry, it helps to improve the aroma of products like teas, confectionery and liquors (*14*). Different extraction techniques have been used to extract these compounds from citrus waste.

To reduce waste, citrus byproducts can be utilised for the production of bioactive compounds and value-added products. The predominant methods for extracting essential oils and extracts usually include hydro- and steam distillation, solvent extraction and cold pressing. However, newer and more environmentally friendly extraction techniques, such as microwave extraction, ultrasound extraction and supercritical fluid extraction, have come into use as they require less energy and solvents, committing to sustainability. These methods are also used for the extraction of other bioactive compounds (*15*). This review provides knowledge on the effective utilisation of citrus waste for the extraction of bioactive compounds. Other compounds are identified, analysed and utilised for their health benefits and use in various industries. The main objective is to reduce waste, minimise pollution and promote a circular economy. In addition, this review presents the potential of citrus waste for conversion into bioactive compounds and value-added products. The methods of phytochemical extraction are not only profitable but also environmentally friendly. This is aimed at further development in innovation and research. By focusing on the latest advancements, this review helps to bridge these gaps by highlighting scalable techniques that minimise the impact on the environment.

## CITRUS WASTE: CHARACTERISTICS AND COMPOSITION

In relative terms, 33 % of the produced citrus fruits are processed, resulting in 50–60 % of organic waste containing peel, pulp and seeds (*16*). Citrus fruit processing generally produces solid (*e.g*. peels, seeds, rags and sludge), liquid (*e.g*. cannery effluents, fruit-washing and wastewater) and distilled effluents (*e.g*. citrus molasses, citric acid and pectin effluents), which are isolated by solid-liquid separation. The most important by-product of citrus fruit processing is citrus peel waste.

The analysis of citrus waste shows remarkable difference in the nutrient composition of peel, pulp and seeds. Citrus peel has a moisture content of (75.30±10.20) %, with high content of crude fibre ((57.0±10.0) %) and crude protein ((10.2±3.7) %). It also contains (2.22±6.10) % crude fat and (3.33±0.50) % total ash. In contrast, citrus pulp contains more moisture ((85.7±0.0) %), but significantly lower amounts of crude fibre ((4.9±0.0) %) and protein ((8.6±0.0) %). The fat and ash content of the pulp are (4.9±0.0) and (6.5±0.0) %, respectively. Citrus seeds, for which data on moisture content is not available, are characterised by a high crude fat content (52.0 %) and comparatively low fibre (5.5 %), protein (3.1 %) and ash (2.5 %) contents (*17*).

Traditional disposal methods of citrus processing waste such as incineration and landfilling are inadequate and complicated with environmental consequences, as these processes produce harmful methane gas, emit a foul odour, consume a lot of energy and have slow reaction kinetics (*18*). The unauthorized disposal of waste from citrus processing can pollute both soil and water bodies. In some cases, it can destroy the aquatic environment, especially if there is not enough water to properly dilute this waste. Recently, various ways of utilising waste from citrus processing have been explored to reduce management costs and prevent environmental damage (*18*). This process is known as ’waste valorization’, where waste is upcycled into new products by converting and improving its value into renewable chemicals, fuels and energy.

Citrus processing waste, together with other vegetable matter, makes good compost. Combining orange peel waste with the organic fraction of municipal solid waste (OFMSW) during composting resulted in a 37 % reduction in odour production, which helps to control soil erosion (*19*). An economical and straightforward approach to managing large quantities of waste produced by citrus processing industry is the incorporation of citrus byproducts into animal feed. Fresh citrus byproducts are rich in carbohydrates and low in lignin, accounting for about 28.5 and 3.5 % respectively. This makes them ideal for the digestive system of ruminants and the fibre content helps to improve the animal nutrition, which in turn improves the meat quality (*20*). These byproducts from citrus waste have sparked scientific interest in developing environmentally friendly solutions as they can be valorized.

A novel approach known as the 'biorefinery' concept has emerged, which emphasises the recycling or reuse of citrus byproducts to meet growing demand. These byproducts and biomass are used to extract bioactive compounds for the creation of value-added industrial products using sustainable methods (*1*). At the centre of this concept is the idea of converting various organic waste streams, including agricultural, industrial and municipal wastes, into a wide range of valuable products, such as biofuels, chemicals and materials (*21*). The biorefinery concept applied to citrus waste involves the integration of different technologies and processes to extract and convert the various compounds contained in the waste into a wide range of products. Citrus peels, for instance, are a rich source of valuable compounds such as essential oils, flavonoids and pectin, which can be extracted and utilised in the development of food, cosmetic and pharmaceutical products. Additionally, the residual biomass can be further processed to produce biofuels, biochemicals and even bio-based materials (*22*).

The key to the success of the biorefinery approach lies in the ability to maximise the valorization of all components of the citrus waste stream. The concept of circular bioeconomy, which emphasises the sustainable and efficient use of renewable resources, is closely linked to the biorefinery approach. By incorporating the principles of the circular bioeconomy, the biorefinery concept can strive for zero waste, where every byproduct or side stream is utilised to generate additional value-added products. Through the integration of various technologies, such as fermentation, hydrothermal processing and extraction, the biorefinery can transform citrus waste into a variety of high-value compounds, contributing to the transition towards a more sustainable and circular economy (*23*).

**Fig. S1:**
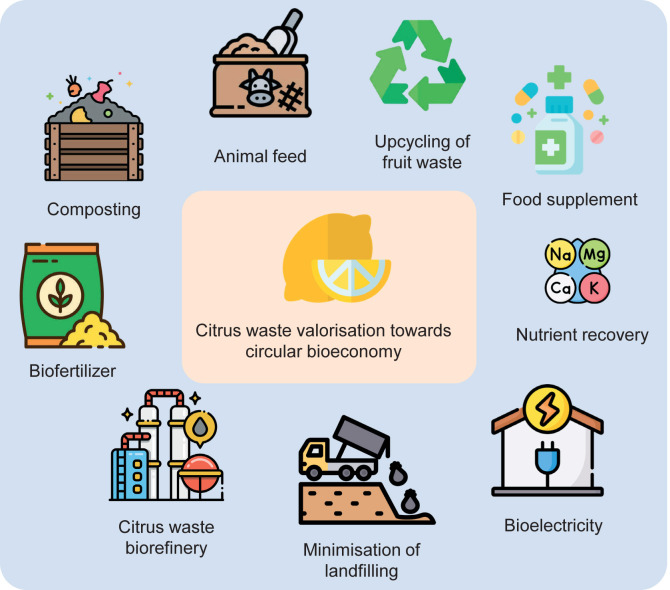
Citrus waste valorization into valuable products and transitioning to a circular economy

The biorefinery concept applied to citrus waste has the potential to address several environmental and economic challenges. By recovering and converting the organic matter in citrus waste into valuable products, the biorefinery can reduce the environmental impact associated with the disposal of this waste, which can otherwise lead to pollution and greenhouse gas emissions. Additionally, the biorefinery can create new revenue streams and employment opportunities, contributing to the economic development of regions with a strong citrus processing industry. The valorization of citrus waste into valuable products demonstrates a concept of waste reuse and recovery that represents a transition towards a circular economy (Fig. S1).

### Extraction technologies of citrus waste

Conventional techniques like Soxhlet extraction, liquid-liquid extraction (LLE), infusion, maceration and solid-liquid extraction (SLE), as well as the extraction of essential oil from citrus peel waste, require high energy input, long extraction times and additional reagents. Researchers investigated microwave-assisted hydrodistillation (MAHD) as a method for extracting essential oil from moist citrus peel waste. This approach reduced costs, eliminated the need for preservatives and improved process efficiency, suggesting that it could be a viable alternative to traditional methods. In the last decade, various integrated technologies including ultrasound-assisted extraction (UAE), microwave-assisted extraction (MAE), pulsed electric field (PEF) and enzymatic hydrolysis have been used to extract and separate valuable compounds such as carotenoids, polyphenols and essential oils from citrus fruit processing (*24*). Physical, thermal, chemical and biochemical processes can be used to convert citrus waste into biofuel (*25*).

Solid-state fermentation (SSF) has been used to extract antioxidant-rich biological components from citrus byproducts such as oranges, lemons, tangerines and grapefruits. d-galacturonic acid is obtained by the hydrolytic action of pectinases on the pectin present in citrus pulp. This compound serves as a key platform chemical for the development of value-added products in citrus waste biorefineries. The use of SSF in such biorefineries significantly reduces the production costs of the pectinases used in this process (*26*). The valorization of citrus peel waste offers great potential for the transition to a bioeconomy. Furthermore, the negative environmental impact of citrus processing industry emphasises the importance of valorization strategies. It is crucial to develop environmentally friendly valorization methods that promote an inclusive biorefinery system to reduce the negative impact on the environment (*16*). MAE is known for its fast processing and high energy efficiency, making it an effective technique for the rapid recovery of bioactive compounds. UAE enables fast extraction through the use of ultrasonic waves, which enhance cell disruption and promote solvent penetration, requiring minimal amounts of solvent. Although supercritical fluid extraction (SFE) involves longer processing times, it offers highly selective and efficient extraction of target compounds with exceptional purity. SFE is also considered environmentally friendly as it utilises non-toxic solvents such as supercritical carbon dioxide. Table 1 shows the extraction technologies used to obtain different bioactive compounds.

**Table 1:** Methods of extraction of different bioactive compounds

Extraction method	Principle	Solvent used	Power/settings	Process	Advantage	Example of parameter for citrus waste
Microwave-assisted extraction (MAE)	Microwaves cause molecular rotation and friction in polar solvents, rapidly heating the solvent and plant material.	Ethanol, methanol, water, acetone-water or ethanol mixtures	*P*=300-900 W, *t*=5-30 min	Citrus waste is dried, ground and mixed with a solvent, heated using microwaves and filtered. Solvent is evaporated to concentrate extracts.	Fast extraction, higher yield, energy-efficient, reduced solvent use	*P*=600 W, *φ*(ethanol)=80 %, *V*(ethanol):*m*(sample)=10:1, *t*=10 min, temperature=60-80 °C, extracted compounds: flavonoids, phenolics, oils
Ultrasound-assisted extraction (UAE)	Ultrasound waves cause cavitation, which creates high energy, disrupting cell walls and releasing compounds.	Ethanol, methanol, water, ethanol-water mixtures	*ν*=20-40 kHz, *P*=300 W, *t*=10-20 min	Citrus waste is dried, ground and mixed with solvent, subjected to ultrasound waves, filtered and solvent is evaporated to concentrate extracts.	Efficient cell disruption, fast extraction, reduced solvent use, low energy consumption, retention of bioactive compounds	*ν*=20 kHz, *P*=300 W, *φ*(ethanol)=70 %, *V*(ethanol):*m*(sample)=10:1, *t*=15 min, temperature=25-30 °C
Supercritical fluid extraction (SFE)	Supercritical CO_2_ dissolves nonpolar compounds; ethanol as a co-solvent increases polarity to extract polar compounds.	Supercritical CO_2_, ethanol as co-solvent	*p*=10-35 MPa, temperature=35-80 °C, *t*=30 min to several hours	Citrus waste is dried, ground, placed in an extraction vessel and subjected to supercritical CO_2_ and ethanol. The extracts are separated and CO_2_ is recycled.	Selective extraction, high purity, eco-friendly, no thermal degradation, broad range of compounds	*p*=25 MPa, temperature=40 °C, solvent: CO_2_ with 5 % ethanol, flow rate=2 mL/min, *t*=1 h, extracted compounds: oils, flavonoids, phenolics

## EXTRACTION OF BIOACTIVE COMPOUNDS FROM CITRUS WASTE

### Processing of citrus waste and extraction techniques for phytochemicals

**Fig. S2:**
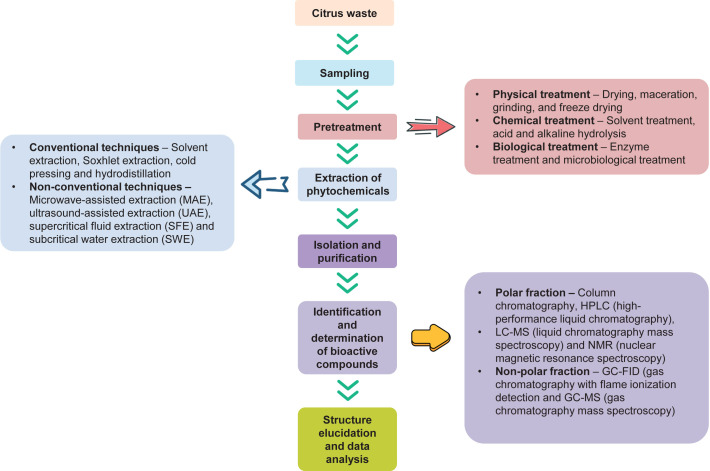
Overview of the processing of citrus fruit waste and extraction of bioactive compounds

Citrus plants are extensively processed fruits and they produce numerous byproducts that are rich in bioactive compounds such as pectins, essential oils and both water-soluble and insoluble antioxidants. Some of this waste is currently being upcycled with different extraction methods that support the idea of waste valorization. This approach not only increases profits but also provides high-quality bioactive compounds. First, it is essential to identify the optimal source of the desired bioactive ingredient, as the quantity and distribution of different compounds vary significantly among citrus species. Although bioactive compounds are typically found in fruits and byproducts of processing, they can also be found in significant concentrations in discarded parts, which effectively harmonises with the goal of waste reduction in numerous scenarios. After the material has been selected, the extraction conditions and techniques must be optimised for each matrix and compound. In this context, not only the quantity of extracted compounds is significant, but also the desired bioactive ingredients. Nevertheless, the purification processes may require the use of solvents that are not environmentally friendly, leading to a considerable increase in overall production costs. After incorporating the extracted compounds into the products, it is essential to evaluate their bioavailability and bioaccessibility, and to consider the interactions between the compounds and other components of the matrix that could affect their availability (refer to Fig. S2) (*27*).

However, some of these extraction methods may have significant drawbacks such as extended extraction time, degradation of the chemicals of interest at high temperatures and health risks (*28*). Table 2 (*8*, *29*-*42*) shows the different bioactive compounds extracted from citrus waste together with their origin, extraction method and their respective functions.

**Table 2:** Bioactive compounds from citrus waste

Bioactive compound	Source	Method of extraction	Best performing solvent	Function	Reference
Limonene	*Citrus sinensis* peel	Solvent extraction	1-ethyl-3-methylimidazolium chloride	Anticancer, antifungal	(*8*, *29*)
Flavonoids	*Citrus reticulata* peel	Pulsed discharge extraction	2-hydroxypropyl-β-cyclodextrin	Antioxidant, anti-inflammatory, antimutagenic, antimicrobial, anti-carcinogenic	(*30*, *31*)
Flavonoids (hesperidin)	*Citrus limon* pomace	Ultrasound-assisted extraction	Water	Antioxidant, antimicrobial	(*32*)
Flavonoids (naringin)	*Citrus paradisi* peel	Supercritical fluid extraction (SFE)	CO_2_+EtOH	Anti-inflammatory, anticancer	(*33*, *34*)
Flavonoids (narirutin and hesperidin)	*Citrus reticulata* peel	Solid-phase extraction (SPE)	Ethanol	Antifungal	(*35*)
Phenolic compounds	*Citrus sinensis* peel	Liquid-liquid extraction	Ethanol	Antifungal	(*36*)
Carotenoids	*Citrus sinensis* peel	Ultrasound-assisted extraction (UAE)	d-limonene	Antioxidant	(*37*, *38*)
Pectin	*Citrus aurantifolia* peel	Microwave heating extraction	Citric acid	Anticancer	(*39*, *40*)
Pectin	*Citrus grandis* peel	Solvent extraction	Deep eutectic solvent (DES) (lactic acid/glucose/water in a ratio of 6:1:6)	Antimicrobial	(*41*, *42*)

#### Sampling

Sampling involves taking samples from different regions. The selection of a sample depends on the climate and the type of product to be recovered. After collecting, the samples are dried or powdered and stored. The sampling methods for citrus fruit waste vary depending on the region, product type and desired result. Among various methods, freeze-drying has proven to be the most efficient for the production of dried powders from citrus fruits such as *Citrus sinensis* and *Citrus aurantifolia*. This technique effectively preserves the bioactive compounds and is therefore ideal for high-quality extraction and further processing. Freeze-drying at –40 °C ensures long-term preservation of the compounds, while air-drying and blending, as used for *Citrus limetta*, are more accessible but may lead to degradation of the volatile compounds. Each method is selected based on the specific goals of the valorization process, such as maximising the recovery of bioactive compounds or facilitating transport for further processing. Sampling of citrus waste varies by source and region. Sweet oranges from the UK were freeze-dried, ball-milled and then stored at −40 °C (*43*). In Bangladesh, *Citrus sinensis* was dried and powdered, stored at 4 °C (*44*). *Citrus aurantifolia* from Thailand was powdered and vacuum dried (*45*). Italian samples of *Citrus reticulata, C. japonica* and *C. clementina* were homogenised with a mortar and pestle and stored at −20 °C (*46*).

#### Pretreatment

The main aim of pretreatment is to remove the structural barriers and make the citrus waste matrix available for chemical or enzyme action. The enzyme action causes hydrolysis, which leads to the formation of sugars. This is to ensure that there are no barriers that could hinder the hydrolysis and that no byproducts are formed. This process also ensures that there is no contamination. Pretreatment can be carried out by physical (drying, maceration, grinding and freeze-drying), chemical (solvent treatment, acid hydrolysis and alkaline hydrolysis) and biological treatment (enzyme treatment and microbiological treatment) (*47*).

#### Physical methods

Drying is a process to remove moisture and there are several drying techniques. Citrus waste can be dried in the sun using solar energy. Although this process is economical, it requires large space, constant monitoring of drying rate and samples are prone to contamination. Hot air drying, also known as oven drying, is carried out by providing constant air flow from the inside to the outside of the waste to prevent contamination. Microwave drying is a modern method that requires less time, but its limitation is the need of high temperature, which could affect or degrade heat-sensitive compounds. In freeze-drying, the moisture content in the citrus waste is crystallised and removed through phases: liquid to solid and then to vapour phase (*48*). Another physical method is ball milling. This method is cost effective and environmentally friendly. The procedure involves increasing the surface area by reducing the particle size. Mill balls grind the citrus waste into powder form with the use of mill equipment (*49*).

#### Chemical methods

Acid pretreatment (*e.g*. sulfuric acid) is effective in disrupting the lignocellulosic structure by cleaving the glycosidic bonds. This leads to conversion of polysaccharides into monosaccharides and oligosaccharides. Pretreatment with dilute acid is beneficial compared to concentrated acid because it is cost effective, economic, environmentally friendly and also does not corrode as much as concentrated acid (*50*). Alkali pretreatment is another chemical method that uses chemicals like NaOH. The action of alkali on the citrus waste causes lignocellulose to swell, partially solubilizing the lignin in the solution and leaving the cellulose intact. A limitation when using alkali is the retention time, *i.e*. the longer the retention time, the more expensive is to recover the solvent. Sonication assisted by alkali pretreatment can also be used to effectively disrupt the lignin and shorten the retention time. The cavitation of bubbles at the biomass interface by sonication treatment ensures faster disruption of the lignocellulosic matrix, especially in combination with alkaline treatment (*51*).

#### Biological treatment

Enzymes like cellulases, pectinases, xylanases and proteases or peptidases are used for pretreatment in the valorisation of citrus waste to degrade structural polysaccharides and proteins and thus facilitate the release of valuable bioactive compounds. Cellulases hydrolyse the cellulose, while pectinases break down pectin into monomeric units. Most of the cell wall and hemicellulose consist of xylan, therefore xylanases hydrolyse xylan. Peptide bonds can be broken down into peptides and proteins with the help of proteases or peptidases. This enzyme treatment is carried out to make the substrate (citrus waste) available to microbes for fermentation (*52*). Similarly, microbes that produce enzymes can be used for pretreatment. Lignocellulosic biomass consists of lignin, cellulose and hemicellulose. *Pleurotus* spp. belong to the white-rot fungi and produce enzymes such as laccases, versatile peroxidases and manganese peroxidases, which are able to degrade lignocellulosic biomass (*53*).

### Extraction of phytochemicals

#### Conventional techniques

Solvents like methanol, water, acetone and ethanol can be used for the extraction of flavonoids, vitamins and phenolic compounds. Oil can be extracted by cold pressing citrus seeds. This technique is environmentally friendly and cost effective, but its disadvantage is lower yields than by solvent extraction. In hydrodistillation, a sample is boiled with water in a flask. The water vapour, together with the volatile citrus compound, is passed into a column and then into a water cooler, where the vapour is cooled and collected as a distillate. Essential oils can be extracted using this method (*54*).

#### Solvents used in extraction

Methanol is a highly polar solvent used for the extraction of phenolic compounds and flavonoids from citrus waste by breaking down cell walls and dissolving polar bioactive compounds. It is particularly effective in extracting various phenolic acids, including those with significant antioxidant properties. Ethanol, another polar solvent, is used to extract flavonoids and phenols and is preferred in the food and pharmaceutical sectors due to its lower toxicity than of methanol. Its efficacy is even higher for extraction of flavonoid glycosides, which are prevalent in citrus peels. Acetone extracts both polar and nonpolar compounds and is therefore suitable for a variety of bioactive components, including flavonoids and carotenoids. Its intermediate polarity facilitates the dissolution of compounds that are only partially soluble in water and other solvents, yielding certain compounds like naringenin. Water, often used in conjunction with other solvents, aids in the extraction of water-soluble bioactive compounds such as vitamin C and certain flavonoids. It also plays an important role in techniques like ultrasound-assisted extraction and microwave-assisted extraction to improve the solubility of compounds in solvent mixtures. Hexane, a nonpolar solvent, is mainly used for the extraction of nonpolar compounds such as essential oils and limonene from citrus peels. Its ability to dissolve hydrophobic components makes it ideal for the extraction of volatile oils.

#### Recent advanced techniques

Microwave-assisted extraction (MAE) is proving to be a highly efficient method for extracting phenolic compounds, with high extraction rates, rapid processing times and good product quality at lower cost. The heat irradiated from the microwave is transmitted through the solvent to the citrus sample, where it absorbs the heat and generates moisture. The moisture evaporates, creating a high vapour pressure. This leads to the breakage of the cell walls and the release of certain bioactive compounds from the sample (*55*). Pulsed electric field (PEF) follows the electroporation of cells. In this technique, an electric field strength of 100 to 300 V/cm for the batch process and 20 to 80 kV/cm for the continuous process is utilized. The electrical force leads to the penetration of the cell membrane, which improves its permeability. This creates hydrophilic pores through which the bioactive compounds can be extracted. In ultrasound assisted extraction (UAE), cavitation bubbles are created and when they collaps they cause shockwaves and inter-particle collision, fragmenting the cellular structure. Sonoporation creates pores in the cell membrane and the structure of the cell membrane is broken down by the shear force created by the cavitation. The bioactive compound is then released and dissolves in the solvent (*56*). A supercritical fluid (SCF) is a substance that exists at temperatures and pressures above its critical point, where the usual distinction between liquid and gas phases does not exist. SCFs can diffuse into solid matrices like gases, so that they can dissolve substances in a similar way to traditional liquids. Since many supercritical fluid extractions (SFEs) take place near the critical point of the fluid, slight changes in pressure or temperature cause significant variations in the density of the fluid. The density-dependent selectivity in SFE processes is responsible for the extraction of phytochemicals (*57*). The different extraction methods and the solvents used are given in Table 3 (*58*-*62*).

**Table 3:** Methods used for the extraction of phytochemicals

Extraction method	Source	Component used for extraction	Extracted phytochemical	*m*(sample)/g	Yield/%	Reference
Solvent extraction	Dry albedo of *Citrus sinensis*	Hot methanol	Hesperidin	16.56	2.8	(*58*)
Soxhlet extraction	Peel of *Citrus limon*	*n*-hexane	d-Limonene	50	3.56	(*58*)
Cold pressing	Seeds of *Citrus latifolia*	-	Seed oil	-	34.4	(*59*)
Hydrodistillation	*Citrus limon*	-	Pectin	100	16.58	(*60*)
Microwave-assisted extraction (MAE)	*Citrus aurantium*	Acetone	Flavonoid	100	16.7	(*60*)
Pulsed electric field (PEF)	Peel powder of *Citrus unshiu*	Water	Narirutin	3	33.6	(*61*)
Ultrasound-assisted extraction (UAE)	Peel powder of *Citrus* ×*sinensis*	Hydroethanolic mixture	Citric acid	100	6.4	(*61*)
Subcritical water extraction (SWE)	Peel powder of *Citrus unshiu*	Methanol	Hesperidin	1	5.0	(*62*)

#### Isolation and purification

The citrus waste is first pretreated and then immersed in a solvent in a specific solvent-to-solid ratio to ensure that all the material is completely covered. The mixture is agitated using a shaker or a stirrer for a predetermined amount of time, usually ranging from several hours to overnight, to allow the solvent to permeate the cell structure and dissolve the target bioactive compounds. After extraction, the mixture is filtered using a filter paper or centrifuged to separate the solid residue from the liquid extract containing the dissolved compounds. The solvent is then evaporated using a rotary evaporator under reduced pressure to concentrate the bioactive compounds, leaving behind a semi-solid or powdered extract. The isolated bioactive components need to be purified for commercial purposes. Crystallography, chromatography, partition techniques, among others, are available for product purification. Different chromatographic methods, including size exclusion, gel filtration partition and adsorption, are available. The chromatography is chosen based on the component that needs to be extracted. Chromatography consists of a stationary phase and a mobile phase to separate the desired components from a mixture of compounds. Thin layer chromatography can detect the presence of a phytochemical. A column chromatography is nothing but a column filled with a stationary phase. Sample is injected at the stationary phase and the separation process occurs through the mobile phase consisting of a solvent (*63*).

High pressure liquid chromatography (HPLC) is considered the best and safe method for obtaining pure phytochemicals. HPLC consists of a solvent delivery pump, an injection valve, an analytical column, a guard column, a detector and a recorder. The citrus sample is introduced into the carrier stream through the injector. The compounds are retained based on the physicochemical interactions between the analytes in the citrus sample and the stationary phase (*64*).

#### Identification and determination of compounds

Identification and determination involve determining the presence of a functional group, multiple bonds and rings, as well as the arrangement of carbon and hydrogen. The methods are as follows. High-performance liquid chromatography (HPLC) is used to separate the molecules in the column, after which the molecules are eluted and detected. The detector detects the molecules based on the physicochemical properties of the compound. This signal is converted into a graphical output called a chromatogram in which the peaks are displayed (*65*). Mass spectroscopy (MS) identifies compounds based on their molecular mass and structure. It includes the detection of functional groups, the presence of multiple bonds and rings, the arrangement of hydrogen and carbon and a complete structural elucidation. The molecules bombarded with electrons are ionized and separated based on their mass to charge ratio and then signals are detected and recorded in the form of peak percentage. Nuclear magnetic resonance spectroscopy (NMR) detects the presence of isotopes of hydrogen, carbon and other elements. It also shows the physical properties of bioactive compounds such as the array and number of carbon atoms (*66*).

Gas chromatography and mass spectroscopy (GC-MS), together with other methods such as liquid chromatography and mass spectroscopy (LC-MS) and fractionation, combine the capabilities of GC and MS (*67*). GC separates compounds, while MS provides precise detection and structural insight. GC is characterised by its simplicity and superior separation capabilities compared to methods like LC. When analysing small, volatile molecules, GC-MS stands out for its unrivalled sensitivity, efficiency and productivity. d-limonene was extracted as the main component from *Citrus maxima* Todarii and is listed in Table 4 (*68*-*71*).

**Table 4:** Methods of identification of *Citrus* compounds

Identification method	Equipment	Source	Compound identified	Reference
HPLC-QToF mass spectrometry	Alliance 2695 HPLC system (Waters Corporation, Milford, MA, USA) coupled to a QToF Premier mass spectrometer	*Citrus australasica*	Flavonoids, phenolic compounds, organic acid, limonoids and phenolic acids	(*68*)
NMR	-	Sweet orange	Naringenin, hesperitin, chrysoeriol, sinensetin, 3,5,6,7,3',4'-hexamethoxyflavone, nobiletin, 5-methoxysalvigenin, 3,5,6,7,8,3',4'-heptamethoxyflavone, 3,5,6,7,4'-pentamethoxyflavone and isosakuranetin	(*69*)
GC-MS	DB-5MS nonpolar column	*Citrus maxima*	*w*(compound)/%: d-limonene 21.72, β-linalool 19.58, 4-carene 7.90, α-terpineol 6.20, neryl acetate 4.64, geranyl acetate 4.70, and γ-terpinene 4.57	(*70*)
LC-MS	Agilent 1290 LC system (Agilent Technologies, Palo Alto, CA, USA) coupled to a time-of-flight mass spectrometer (Agilent Technologies)	*Citrus reticulata*	Ferulic acid, nobiletin, 3,5,6,7,8,3',4'-heptamethoxyflavone, kaempferol and hesperidin	(*71*)

### Bioactive compounds and their uses

#### Flavonoids

Flavonoids are found naturally in plants as secondary metabolites that have important biological properties. They are essential for the protection of plants from ultraviolet (UV) radiation (*72*). More than 60 types of flavonoids are present in various citrus plants, which are divided into flavonols, flavones, flavanones, polymethoxylated flavones, flavanonols and anthocyanins. Flavanones account for over 95 % of the total flavonoid content in the flavonoids derived from citrus fruits. Recent findings have proven that the peel of most citrus fruits contains a significantly higher concentrations of flavonoids than the pulp, with the concentrations decreasing as you approach the flesh (*73*). The citrus peels are considered to be the source of polymethoxyflavones (PMFs), notably tangeretin, nobiletin and sinensetin. A method used to extract flavonoids is polarity-dependent extraction. In this process, a deep eutectic solvent (DES), a green solvent, is used. Extraction is carried out with ultrasonic irradiation, with DES. This process yielded higher extracted amounts of total flavonoids (65.8 mg/g) (*74*).

Flavonoids are used as food preservatives as well as colouring and flavouring agents. Citrus flavonoids have diverse biological effects such as antibacterial, antioxidant, anti-inflammatory and antiviral properties. Recent studies have suggested that they can also reduce the risk of cancer, type 2 diabetes, neurological disorders and osteoporosis. Flavonoids contribute to heart protection by mitigating oxidative stress and inflammation, inducing vasodilation and modulating apoptotic processes in the endothelium. Moreover, they interact with lipid metabolism and inhibit platelet aggregation, thereby helping to prevent various cardiovascular diseases (*75*).

#### Phenolic compounds

Phenolic compounds are found in various parts of plants, including fruits, stems, seeds, leaves and roots. In these plant structures, all phenolic compounds contain at least one aromatic ring with a hydroxyl group (*76*). Many phenolic components have been reported to be effective antioxidants, anticancer, antibacterial, cardioprotective, anti-inflammatory and immune system boosting agents. They also protect the skin from UV radiation and are an effective ingredient for medical and pharmaceutical purposes (*77*). Due to their structure, which contains a hydrogen atom and/or an electron, phenolic compounds act as antioxidants and thus disrupt the oxidation chain reaction of free radicals. Oxidative and nitrosative stress in organisms can lead to the production of free radicals, which attack cells and contribute to the development of various diseases such as atherosclerosis, cardiovascular disease, neurological disorders, cancer, hypertension and diabetes mellitus (*78*). Most phenolic compounds occur in nature as mono- and polysaccharide conjugates containing one or more phenolic groups. Additionally, 277 phenolic compounds can be converted to esters and methyl esters. The structural diversity of phenolic compounds leads to a broad spectrum of such compounds found in nature. Currently, 8000 different structures of phenolic compounds have been identified. Polyphenols are involved in the regulation of diameter growth, pigmentation and defense against pathogens. They also serve as signalling molecules to recognize symbionts. As natural antioxidants, polyphenols have important properties such as the inhibition of carcinogenesis and lipid peroxidation, as well as an antibacterial activity. They also act as naturally occurring phytohormones and directly constrict the capillaries. In addition, polyphenols stabilise ascorbic acid and have various other beneficial effects (*79*). Various extraction methods are used for the extraction of phenolic compounds, including conventional solid-liquid extraction (SLE), subcritical water extraction and liquid-liquid extraction (LLE), or accelerated solvent extraction (ASE), microwave-assisted extraction (MAE), supercritical fluid extraction (SFE), ultrasound-assisted extraction (UAE) and pressurized liquid extraction (PLE). The Soxhlet device is used in the traditional SLE. Solvent evaporation is a straightforward process in which the extract and an organic solvent are separated because of their different boiling temperatures, resulting in the isolation of the solvent and the final product in their purest forms (*80*). The extraction of total phenolic content from Persian lemon waste using deep eutectic solvents (DES) resulted in yields, expressed as gallic acid equivalents, between 1.28 and 2.90 mg/g. Among the DES tested, [Ch]Cl:Gly 1:2 showed the highest yield of 2.90 mg/g (*81*). In ultrasound-assisted extraction, phenolic compounds are extracted from Persian lemon waste using a 130-W ultrasonic liquid processor. This processor is equipped with a 13 mm diameter probe and operates at a frequency of 20 kHz (*82*). In microwave-assisted extraction, the interaction of polar molecules like water with the waves destroys the citrus residues due to an increase in pressure and heat inside the cell walls. Because of the rupture of cell walls, molecules are transferred more easily, which increases the extract yield without affecting the chemicals (*83*).

#### Limonoids

Limonoids belong to the family of phytochemicals found predominantly in citrus fruits like lemon, orange, mandarin, grapefruit, lime and bergamot. Limonoids are secondary polycyclic metabolites that are rich in oxygen and are chemically associated with terpenoids (*4*). Citrus fruits have so far produced 17 different forms of limonoid glycosides and 36 different types of limonoids. Numerous citrus fruit seeds contain limonin and nomilin at a mass fraction of around 6 mg/kg (*84*). The two categories of limonoids are limonoid glucosides and limonoid aglycones. Limonoid aglycones, which contribute to the bitter taste of citrus fruits and juices, are predominantly found in citrus peels and seeds, accounting for 80 and 70 % of the total mass, respectively. Through enzymatic activity by limonoid glucosyltransferase, bitter limonoid aglycones are converted into tasteless limonoid glucosides. These glucosides, soluble in water, are mainly found in citrus fruit pulp and juice, comprising 61 and 76 % of the total mass, respectively (*85*). Despite this, citrus juices eventually develop significant bitterness after processing, a condition known as delayed bitterness, which has a detrimental impact on the standard and acceptance of a fruit juice. As a result, many physical, chemical and microbiological processes for debittering citrus juices have been developed to improve their quality and consumer acceptance. Benzene is used to extract limonoids, petroleum ether is used to precipitate them and dichloromethane is used to crystallize them. Benzene is hazardous, so its use has been limited and forbidden in recent years.

Limonoid aglycones are low-polarity chemicals that are generally not soluble in water and they can be extracted with organic solvents using the reflux techniques. After extraction, limonoid aglycones are separated using open-column chromatography. The process then involves fractionation using silica gel chromatography, followed by additional purification using HPLC. Subsequently, spectroscopic techniques are used to analyse the obtained results. In contrast, limonoid glucosides are polar molecules that are obtained using polar solvents. Recently, new methods for extracting limonoids from citrus waste, such as hydrotropic extraction and supercritical carbon dioxide (SC-CO_2_) extraction, have been developed (*86*). Supercritical carbon dioxide (SC-CO_2_) extraction is good for the environment because the use of organic solvents is avoided. The cost of energy necessary to generate high pressure, on the other hand, restricts its practical applicability. The efficiency of a flash extraction technique for the large-scale production of limonin from *Citrus reticulata* Blanco seeds was investigated. Using this method, a limonin yield of 6.8 mg/g with a purity of 95 % and a limonin recovery yield of 97.1 % were obtained (*87*). Limonoid aglycones and glycosides have been shown to have a wide range of pharmacological benefits, such as anticancer, antioxidant, antibacterial, antidiabetic and insecticidal properties (*88*). Citrus limonoids are considered to have anticarcinogenic properties because of their capacity to induce phase II enzymes. The phase II enzymes [nicotinamide adenine dinucleotide (NADH): quinine reductase (QR) and glutathione S-transferase (GST)] play a role in the removal of harmful metabolites. Various limonoids from citrus fruits such as limonene, limonin, nomilin, defuran limonin, deacetyl nomilinic acid glucoside, deacetyl nomilin, limonin glucoside, deacetyl nomilinic acid glucoside, limonin-7-methoxamine and isoobacunioc acid have been shown to induce NADH:QR and GST (*89*). Chemically induced neoplasia has also been observed to be inhibited in the foregut, small intestine, oral cavity, colon, skin and lungs of animals. In addition, it inhibits breast cancer cells (BCCS) from proliferating *in vitro* (*7*).

#### Alkaloids

Alkaloids are primarily indole ring derivatives. They are categorised as heterocyclic (typical) or non-heterocyclic (atypical) metabolites based on their skeletal structure. Alkaloids are abundant in citrus species and are more abundant in orange and lemon peel than other phytochemicals (*90*). In dried citrus peels, synephrine is the most prevalent alkaloid. Octopamine, tyramine and *N*-methyltyramine are other alkaloids isolated from *Citrus aurantium.* Synephrine has a molecular formula of C_9_H_13_NO_2_ and is a sympathomimetic alkaloid that is present as a primary constituent in bitter orange extracts (*86*). About 90 % of citrus phytoalkaloids are composed of it. Water and ethanol extracts can be obtained from the dried and unripe fruit of *Citrus aurantium.* Acriquinoline B and acriquinoline A have also been extracted from *Citrus reticulata* in a similar manner (*91*). The *o*-synephrine, *m*-synephrine and *p*-synephrine are the three isomeric forms of synephrine. Young fruits have the highest content of *p*-synephrine, its concentration decreases as the fruit matures. Its concentration ranges from 53.6 to 158.1 g per litre of juice, and its mass fraction from 1.2 to 19.8 mg per gram of dried fruits and from 0.20 to 0.27 mg per gram of citrus pulp. Synephrine is extracted from aqueous extracts with a strong cation-exchange phase using solid-phase extraction and then derivatized with appropriate reagents. The *p*-synephrine derivative is analyzed using chromatographic and spectroscopic methods like nuclear magnetic resonance (^1^H and ^13^C NMR), GC–MS, GC–FID, and LC-MS (*92*).

Alkaloids, prominent secondary metabolites in citrus fruits, exert various beneficial effects on human health, such as neuroprotection, anticancer properties, antioxidant activity and cardiovascular protection. For thousands of years, dried citrus peels have been used in China as an excellent remedy for asthma (*93*). Recent studies have found that the alkaloid fraction of dried citrus peel has anti-asthmatic properties. The alkaloids have emetic, anticholinergic, anticancer, anti-hypertensive, sympathomimetic, myorelaxant, anti-viral, diuretic, hypnoanalgesic, anti-depressant, anti-tussigen, anti-inflammatory and antimicrobial properties. The *p*-synephrine is also a stimulant that has been shown to have cardiovascular benefits as well as favourable effects on athletic performance and energy expenditure, carbohydrate mobilisation and hunger control, fat oxidation and mass loss, mental alertness and cognition (*94*, *95*).

#### Carotenoids

Carotenoids, a group of isoprenoid metabolites, are widely distributed and synthesised by various photosynthetic organisms, including *Cyanobacteria*, algae and plants, but also by certain non-photosynthetic organisms such as fungi, bacteria, archaea and animals. The presence of carotenoids is responsible for the yellow, red and orange colours observed in a variety of fruits and vegetables (*73*). Carotenoids are broadly classified into two main groups: hydrocarbon carotenoids, often referred to as carotenes (*e,g,* β-carotene and lycopene), and oxygenated carotenoids, commonly known as xanthophylls (such as β-cryptoxanthin, violaxanthin and lutein). Citrus fruits have been found to contain more than 115 carotenoids and their isomers, which contribute to their brilliant and appealing colours. Citrus peels are rich in carotenoids such as β-carotene, β-cryptoxanthin, zeaxanthin, α-carotene and lutein (*96*). β-carotene serves as a colouring agent in commercially produced foods such as dairy products, pasta, margarine and confectionery (*7*). Surpassing, The demand for carotenoids exceeds the 2000-million-dollar market and is increasing steadily. The global carotenoid market is expected to grow at an annual rate of 4 % from 2018 to 2023 (*97*).

Due to their hydrophobicity, carotenoids are traditionally extracted using organic solvents (*98*). Nonpolar solvents such as hexane, tetrahydrofuran or petroleum ether are typically used for the extraction of esterified xanthophylls or nonpolar carotenes. Conversely, polar solvents like ethyl acetate, ethanol and acetone are more suitable for the extraction of polar carotenoids (*99*). Different protocols for the extraction of carotenoids from natural sources are accelerated solvent extraction (ASE) (also called pressurized liquid extraction (PLE)), supercritical fluid extraction (SFE), pulsed electric field (PEF) assisted extraction, enzyme-assisted extraction (EAE) and atmospheric liquid extraction (ultrasound-assisted extraction (UAE) or with Soxhlet, maceration and microwave-assisted extraction (MAE)). One of the main uses of the peel as citrus waste is to extract the carotenoids. Forty terpenoid carbon bonds (double and single) connected by the core of the molecule form its structure. Because of their molecular structure, certain carotenoids have a greater affinity for polar solvents like acetone, while others for nonpolar solvents such as hexane. However, these solvents pose a significant challenge due to their harmful nature and difficulties in disposal. ’Green’ methods such as the UAE treatment have been developed using environmentally friendly solvents for carotenoid extraction (*83*).

Ultrasound-assisted extraction (UAE) resulted in a 40 % higher carotenoid extract than conventional extraction (*37*). Ionic liquid combined with ultrasound-assisted extraction used 1-butyl-3-methyl-imidazolium-chloride [BMIM][Cl] as an ionic liquid to extract carotenoids from citrus peels. This method resulted in a higher total carotenoid content than traditional acetone extraction (*73*). In the food industry, the extraction of valuable nutrients and beneficial compounds from fruit byproducts remains a major challenge. To improve extraction and maximise the extraction of carotenoids, green extractions with GRAS solvents can be a useful alternative (*98*).

## VALUE-ADDED PRODUCTS

### Single-cell protein

Microorganisms cultivated in a dead and dehydrated state on various carbon sources and serve as protein supplements are known as single cell proteins (SCP) (*100*). SCP consist of carbohydrates, fats, vitamins, nucleic acids and minerals and contain about 60 to 82 % protein on dry cell mass. They serve as a source of many essential amino acids as they contain lysine and methionine, which are limited in most plant and animal foods. SCP are a nutritional supplement that can be used as a substitute for more expensive sources such as beans and fishmeal. Citrus waste contains various nutrients and therefore has great potential as a raw material for the production of SCP by fermentation (*101*). The different products from different sources of citrus waste are listed in Table 5 (*25*, *102*-*111*).

**Table 5:** Products obtained from citrus waste

Source	Pretreatment	Process	Product obtained from citrus waste	Reference
Feedstock citrus pulp	Steam explosion	Fermentation	Animal feed	(*102*, *103*)
Citrus residue	Electrofluidic pretreatment	Distillation	Essential oil	(*104*, *105*)
Citrus biomass	Acid-catalyzed steam	Gasification	Biofuel	(*25*, *106*)
Citrus peel in combination with leaf extract	Alkaline peroxide-assisted and hydrothermal pretreatment	Solvent evaporation	Food coating and biodegradable plastic packaging	(*107*)
Citrus peel	Acidification with mineral or organic acids	Milling enzymatic inactivation extraction. vacuum impregnation, supercritical fluid extraction	Gelling agent	(*108*)
Citrus waste produced from juice extraction	Drying	Spray drying emulsification	Encapsulating agent	(*109*)
Citrus peel	Chemical activation using orthophosphoric acid	Pyrolysis	Activated carbon	(*110*)
Citrus seed	Drying	Sodium methoxide-catalyzed transesterification	Biodiesel	(*111*)

SCP were produced by fermenting orange waste using *Saccharomyces cerevisiae* and *Aspergillus niger* as microbial sources and contributed to the efficient valorisation of citrus by-products. SCP were obtained by batch cultivation using orange waste from species including *Citrus aurantium, Citrus sinensis* and *Citrus paradisi* as the substrate. The crude fibre, crude fat, ash and protein in the fungal biomass were estimated for each batch and it was found that the protein content of *A. niger* (29.0, 29.75 and 27.15 % for *C. aurantium*, *C. paradisi* and *C. sinensis*, respectively) was increased compared to *S. cerevisiae* (23.50, 22.50 and 22.06 % for *C. paradisi*, *C. sinensis* and *C. aurantium*, respectively). The high monosaccharide content of *A. niger* led to an increase in protein content compared to *S. cerevisiae*. SCP had low crude fat, crude fibre and ash content (*112*).

SCP were produced by *S. cerevisiae* in submerged fermentation with different media like fruit hydrolysate medium, which contains only components released from the fruit waste and is therefore rich in glucose and other nutrients that promote microbial activity and protein production. A high percentage (60.31 %) of protein was obtained when grown on supplemented fruit hydrolysates. The increase in yield was due to the presence of a carbon source like glucose. The medium with fruit hydrolysate provided 53.4 % of protein, while the medium with supplemented fruit hydrolysate provided the lowest yield of 17.47 %. This indicates that a low nitrogen supply leads to lower SCP production (*113*).

#### Pectin

Pectin is a complex polysaccharide present in plant cell walls and is often found in citrus waste and other byproducts of fruit and vegetable processing. It consists mainly of a lengthy linear polymer of α-1,4-glycoside-linked d-galacturonic acid known as homogalacturonan. The content in mature fruits is from 3 to 7 % on dry mass basis and 0.1 to 1.1 % on fresh mass basis. Pectin can be used as a bulking and coating agent, as well as viscosity modifier, chelating agent, water-soluble biodegradable film and emulsifier (*8*). It is a gelling and thickening substance used in the food industry. Detoxification, blood glucose reduction and anti-diarrheal properties are some of its medicinal uses. Pectin is used for various purposes, including lowering blood lipoprotein levels, as an agglutinant in blood therapy, to treat high triglyceride and cholesterol levels, and for the possible prevention of prostate and colon cancer. It is also considered for the treatment of gastroesophageal reflux disease (GERD) and diabetes, as well as for its potential to prevent poisoning from heavy metals such as lead and strontium (*114*). It may also help to reduce the risk of heart disease and gallstones.

Manosonication extraction was carried out with the peels of *Citrus unshiu* to extract pectin enriched with rhamnogalacturonan-I (RG-I). The physicochemical and macromolecular properties of pectin have been studied. Compared to conventional maceration extraction of pectin (18.3 %), manosonication extraction showed a higher yield (25.5 %) (*115*). Pectin was extracted from the citrus pomace biomass using a mild organic acid like acetic acid. A high volume fraction of acetic acid (9 %) was used and preliminary experiments were carried out with citrus biomass for 2, 4 and 6 h. A higher extraction yield was observed with a treatment for 6 h (23.7 %) than with a treatment for 2 h (16.4 %). Using a more environmentally friendly method, like MAE, to extract pectin from *Citrus limetta* peel is an efficient way to turn waste into a useful food additive. Under optimal conditions, *i.e.* 600 W microwave power, pH=1 and a duration of 180 seconds, the highest yield of 32.75 % was achieved. FTIR and ^1^H NMR spectra were used to validate the integrity of the pectin skeleton (*116*).

### Essential oils

Essential oils are aromatic compounds found mainly in oil glands or sacs distributed throughout the fruit peel, especially in the cuticles and the outer coloured layer known as the flavedo. They are insoluble in water but soluble in ethers, natural oils and alcohols. Citrus essential oils are a sustainable, natural alternative and environmentally friendly compared to chemical preservatives and other synthetic antioxidants commonly used in food preservation, like sodium nitrates, nitrites or benzoates (*86*). Citrus-based essential oils are derived mostly from citrus peels, which are usually discarded as waste and contribute to environmental problems. Citrus oils extracted from discarded peels are not only environmentally friendly, but can also be used in a variety of applications, including food preservation (*7*).

Citrus essential oils contain about 200 components, including sesquiterpenes, terpenes, alcohols, esters and aldehydes, and can be defined as a mixture of oxygenated chemicals, terpene hydrocarbons and non-volatile residues. Terpenes are unsaturated compounds that degrade rapidly when exposed to light, heat and oxygen. To mitigate undesirable flavours, terpenes are often eliminated. They make up approx. 80-98 % of most citrus peel oils (*117*). Limonene is the most volatile component of citrus essential oils and plays a crucial role in determining their chemical, physical and biological properties. Its amount in essential oils varies depending on the variety and ranges from 32 to 98 %. For instance, lemon essential oil typically contains 45-76 % limonene, sweet orange essential oil contains 68-98 % limonene and bergamot essential oil contains 32-45 % limonene (*118*).

Cold pressing is the conventional method of extracting essential oils from citrus peel. It removes cuticle oils and peels mechanically, leaving a dilute emulsion that is then centrifuged to extract the essential oils (*119*). Simple distillation and steam stripping processes have been found effective in extracting oil components from oil mill sludge. When citrus fruit peels are exposed to boiling water or steam evaporation, essential oils are released by distillation. The essential oil vapours condense and are collected in a separate chamber called a florentine flask (*86*). Essential oil was extracted from lemon peel waste by microwave-assisted extraction with a yield of (2.0±0.2) %. The results showed the essential oil composition and detected the presence of 65.1% limonene, 14.5 % β-pinene and 9.7 % γ-terpinene (*120*).

### Activated carbon

Activated carbons with a high methyl orange adsorption capacity were also produced from the residue after pectin extraction and H_2_PO_2_ activation. Many chemical activating agents, such as zinc chloride and potassium hydroxide, are very corrosive and hence harmful to the environment. Due to its low corrosive effect and non-toxicity, K_2_CO_3_ is considered environmentally friendly. It has also proven to be a highly effective activating agent for the production of activated carbon. Thus, activated carbon from citrus waste is suitable for the production of high-performance supercapacitors, the removal of metal ions from wastewater and the adsorption of pesticides from water (*121*).

*Citrus limetta* peel waste was effectively utilised for the production of FeCl_3_-activated carbon adsorbents (AC-CLPs) for the removal of fluoride from groundwater and biowaste. The peel biomass was impregnated with FeCl_3_ and then carbonised at two different temperatures, 250 and 500 °C, to obtain different adsorbents. The efficiency of the adsorbents to adsorb fluoride was analysed. Interestingly, a slight increase in the initial fluoride concentration (~0.0025 %) was observed, while the fluoride removal efficiency decreased significantly (from 94.7 to 5.0 % and from 94.8 to 33.3 %). This indicates a temperature-dependent variation in adsorbent performance, possibly due to the changes in structure and surface chemistry caused by higher carbonisation temperature (*122*).

### Citrus waste packaging films

With the increase in food consumption, the demand for food packaging has also increased. Currently, petrochemical-based plastic films are widely used as packaging materials due to their wide accessibility, water vapour permeability, heat sealability and good mechanical strength. However, these plastics pose disposal problems as they are made from non-renewable resources and are not biodegradable. Food is often contaminated by the toxic chemicals contained in the packaging material. Given the problems with plastic packaging, the development of biodegradable packaging is not only a practical necessity, but also an important ecological requirement (*123*).

Pectin is abundant in citrus peel. Different reinforcing agents can be used to produce edible pectin-based films. It is a water-soluble component that polymerizes into films when a film-forming solution is cast and dried. Pectin films have high mechanical strength and barrier properties (oils and oxygen), but they have a weak moisture barrier. Cellulose from citrus fruit waste is used as an alternative to glass fibre and can provide mechanical strength to biodegradable and biocomposite films (*124*). The physical properties of the biodegradable film based on orange waste are similar to those of commercially available plastic. In addition, the produced film had good antibacterial, thermal and mechanical properties (*125*). Packaging films are considered good materials if they have antibacterial, biodegradable and antioxidant properties (*126*).

Recent research shows the advantages of utilising citrus waste by producing edible films based on grapefruit pectin. This innovative film offers various benefits, such as antimicrobial properties for food packaging, extending the shelf life of perishable goods and serving as environmentally friendly alternative to synthetic packaging materials. The oxidation of lipids in food can be controlled by antioxidants. They are therefore used to improve the shelf life and the nutritional value of stored food. Bio-based high-density polyethylene (bio-HDPE) films have been developed using phenolic compounds found in citrus waste. Extrusion was used for melt-mixing each natural additive (0.8 parts per hundred resin) of bio-HDPE, and the resulting pellets were then thermally compressed as films (*127*).

### Food-grade kraft paper

Kraft paper is commonly used for packaging flour, sugar, dried fruits and vegetables (*128*). Utilising renewable energy sources for food coating and packaging is imperative on a global scale, as opposed to relying solely on synthetic materials. However, paperboard and paper are biodegradable and have greater mechanical strength, which is unsuitable for packing foods with high moisture content because they may contain pores through which moisture and some gasses can pass. The growing concern about plastic waste accumulation has sparked interest in developing biodegradable materials with moisture barrier properties. Studies have found that incorporating wax or zein into kraft paper can effectively improve its quality by creating vapour barrier. The water permeability of a composite film made with lipid-hydroxypropyl methyl, cellulose and wax is significantly reduced (*17*).

Biological materials with hydrophobic properties, like citrus wastes, improve water barrier properties while avoiding the weakening of mechanical properties and, ultimately, destruction. The leaf extract (10 mL) has been used to treat the kraft paper (terpene and limonene hydrocarbons). The solvent was then evaporated completely at 25 °C during 24 h using a mechanical shaker (at 60 rpm). The components of the extracts were partially dissolved in the available space and dispersed among the cellulose fibres of the paper. This process resulted in the formation of a thin layer of extracts on the paper surface. Limonene is a mixture of isoprenoid-related chemical compounds that are extracted in large amounts by distilling the oil from citrus peels. The strength of kraft paper was improved with the application of a coating derived from dissolved expanded polystyrene (EPS) waste in limonene (*129*).

### Biopolymers

Biopolymers are produced through intricate cellular metabolic pathways facilitated by enzyme-driven polymerase chain reactions. These biodegradable polymers find extensive application in various industries, with a notable emphasis on medical fields. Natural biodegradable polymers include polysaccharides like chitosan, cellulose derivatives and starch, as well as protein-based polymers such as albumin, collagen and gelatin. Synthetic biodegradable polymers, on the other hand, comprise poly(amino acids), aliphatic polyesters, phosphorus-based polymers, poly(alkyl cyanoacrylates), polyanhydrides and acrylic polymers. Biopolymers can be produced from food waste by extraction or fermentation, regardless of pretreatment, to obtain fermentable sugars (*130*). Biopolymer scaffolds stimulate the regeneration of bone tissue and promote cell adhesion, differentiation and proliferation. There are two types of biopolymers used as scaffolds: natural and synthetic. Using synthetic polymers offers benefits such as improved elasticity, degradation rate and mechanical strength. Conversely, natural scaffolds show favourable properties in interacting with host cells and tissues, have lower immunogenicity and significant bioactive potential. d-limonene, found in citrus waste, is used as a flexible feedstock for the production of biopolymers. Tert-butyl hydroperoxide (TBHP) is used to oxidize it, yielding limonene oxide and tert-butyl alcohol (TBA). When CO_2_ and a catalyst β-diiminate zinc acetate complex co-polymerize, poly(limonene carbonate) (PLC) is formed (*131*).

The production of biopolymers was investigated in mixed microbial culture. The wastewater from citrus processing industry was used to produce biopolymers in a membrane bioreactor (MBR) system. Biopolymers were also produced using acetate and the rate of acetate conversion to biopolymers was determined. When CO_2_ and a catalyst diiminate zinc acetate complex co-polymerize, PLC is formed. The mass fraction of biopolymer was found to be 0.56 mg/mg when fermented citrus wastewater was used. The results show that citrus wastewater can serve as a cost-effective substrate, with a productivity rate of 56 %, comparable to that of acetate at 55 % (*132*).

### Bioenergy

Citrus waste has been recognised as a viable biomass reservoir for the production of renewable energy and liquid fuels. Within the renewable energy sector, bioenergy accounts for the largest share. Various methods have been developed and simulated, including gasification, pyrolysis, grate firing, biogas plants, hydrothermal liquefaction, quad-generation plants and biogas upgrading, to produce electricity from biomass (*133*). The physicochemical properties of citrus waste make it suitable for anaerobic digestion, a process that reduces its organic content and produces valuable products such as biogas. By combining the energy from solid waste burning with the energy from anaerobic digestion, enough steam could be generated to meet all of the process steam and power needs of the facility. According to a recent study, citrus waste de-oiled after the essential oil extraction could produce 322.6 mL/g volatile solids at a Fenton dosage of 30 % (*134*).

### Biofertilizer

The irrational disposal of fruit and vegetable waste (FVW) is all too common and poses a significant environmental and economic risk. The conversion of vegetable and fruit waste into biofertilizers through anaerobic digestion has the potential to reduce pollution while improving soil nutrition. The main problem in bioprocessing FVWs is the high carbon and low nitrogen content (*135*). Bbiofertilizers can be produced from citrus processing waste by changing the pH, C/N ratio and water content of the waste to 6.3, 24:1 and 60 %, respectively. Animal manure has been used in a batch process for the anaerobic digestion of olive and citrus industry waste. The composition of the produced biofertilizer (digestate) was significantly related to the feedstock content. The results suggest that a specific mixture of animal dung, citrus pulp and maize silage is required to produce significant volumes of biogas. Additionally, the resulting digestate, which is rich in antioxidants, can be effectively used as a fertilizer in agriculture (*136*).

#### Biofuels

Biofuels are renewable fuel alternatives for reducing anthropogenic greenhouse gas emissions. Compared to thermochemical conversions, the byproducts of citrus waste are much more suitable for biofuel production through biochemical conversions, as these citrus wastes have high moisture content. The production of biomethane from citrus byproducts is reported to yield between 300 and 600 mL of methane per gram of volatile solids, while the yield of bioethanol is from 50 to 60 litres per tonne of citrus waste (*24*). When biofuels are compared to petroleum fuel or gasoline, they are expected to reduce carbon dioxide emissions by as much as 80 % (*137*). About 70 % of citrus waste consists of carbohydrates and could produce 1200 million litres of ethanol worldwide. A horizontal fixed-bed pyrolysis reactor (FBR) was used for slow pyrolysis and torrefaction experiments in the temperature range of 200–650 °C to extract bio-oil and charcoal from orange and lemon peel waste (*138*). Pyrolysis of the peel residues in the temperature range of 400–650 °C leads to the production of high-energy biochar and tars (*139*). Of all bio-based fuels, biodiesel is categorised as a type of fuel that is used as a permanent replacement for diesel. The high oil content of 27-52 % in bitter orange seeds makes them an economically viable and reliable source for biodiesel production (*140*). The lemon peel oil (LPO) can be used as a substitute for diesel. It has been used to reduce NOx and smoke emissions. Four emulsified fuel samples were made with different stability and brake thermal efficiency (*141*).

## VALORIZATION OF BYPRODUCTS

The valorization process, which aims to maximise the value and utilisation of agricultural and industrial byproducts, involves a complex interplay of different stakeholders, each with their own perspectives, goals and challenges. Farmers, for instance, are often the primary producers of these byproducts and seek to find cost-effective and sustainable ways to manage and repurpose them (*142*). Meanwhile, industry players, such as food and beverage manufacturers, are interested in utilising the potential of these byproducts as high-value ingredients or materials for their own products, contributing to a more circular economy. Policymakers, on the other hand, play a crucial role in shaping the regulatory framework, promoting sustainable practices and creating an environment that enables the successful valorization of byproducts. Farmers, as the first link in the valorization chain, face the challenge of managing the vast quantities of byproducts generated by their agricultural activities. These byproducts, previously considered as waste, can now be transformed into valuable resources through the biorefinery concept, where they are repurposed into biomaterials, biochemicals and biofuels (*143*). This shift in perspective not only offers farmers additional sources of income, but also contributes to the overall sustainability of their operations by reducing waste and environmental impact.

Industry players, on the other hand, are increasingly recognising the potential of these byproducts as high-value materials for their own products. The use of food waste and other byproducts as feedstock for the production of microbial oils, carotenoids and other valuable compounds is in line with the concept of circular bioeconomy, which aims to minimise waste and maximise the value of available resources (*144*). Policymakers play a crucial role in facilitating the valorization process by creating a regulatory environment that incentivizes sustainable practices and enables the successful commercialization of valorized products. Integrated product policies, such as those implemented in the European Union, create market-based incentives for companies to engage in a continuous supply and demand of valorized products, thus encouraging the adoption of valorization practices in different industries. The successful valorization of byproducts requires the active cooperation and coordination of these different stakeholders. Farmers, industry players and policymakers need to work together to overcome challenges, share knowledge and develop innovative solutions that improve the viability and social acceptability of the circular bioeconomy (*23*).

### Life cycle assessment of citrus waste valorization

Researchers have investigated various valorization processes to recover and utilise the valuable compounds from citrus waste. These processes can be broadly classified into three main categories: extraction of specific compounds, conversion of waste into biofuels, and development of value-added products such as food additives, dietary supplements and packaging materials. The extraction of specific compounds from citrus waste is an important focus of recent research. The outer peel of citrus fruits can be divided into two areas, the flavedo and the albedo, each containing different bioactive compounds. These compounds can be selectively extracted and purified for use in pharmaceutical, cosmetic and other high-value applications (*145*).

In addition to compound extraction, citrus waste can also be converted into biofuels, such as bioethanol and biogas, through fermentation and anaerobic digestion. These processes not only valorize the waste, but also reduce the environmental impact of waste disposal. In addition, citrus waste can be used to develop a variety of value-added products, including dietary fibre, pectin and natural food colourants and preservatives. These products can be incorporated into a wide range of food, pharmaceutical and cosmetic formulations and provide a sustainable and cost-effective alternative to synthetic ingredients (*86*).

The valorization of citrus waste is a complex process that requires a comprehensive life cycle assessment to ensure the economic, environmental and social sustainability of the different valorization strategies. Such assessments should consider the entire life cycle of the valorization process, from the collection and transport of the waste to the production, distribution and end-use of the recovered products. By implementing a circular economy approach to citrus waste valorization, the environmental impact of waste disposal can be reduced while creating new revenue streams and promoting sustainable development (*146*).

### Industrial application of citrus waste valorization

One industry that has already embraced the potential of citrus waste is the agricultural sector. Citrus peel, which makes up nearly 50 % of fresh fruit mass, can be used as a natural source of antioxidants, dietary fibre, enzymes and organic acids, as well as soil amendments and animal feed supplements. The cosmetics and personal care industry has also capitalized on the unique properties of citrus waste. The essential oils and bioactive compounds extracted from citrus peels and seeds have been incorporated into a variety of products, ranging from fragrances to skincare formulations, utilising their antimicrobial, anti-inflammatory and anti-ageing properties (*86*). Furthermore, the pharmaceutical industry has recognised the potential of citrus waste-derived compounds as sources of natural antimicrobials, anti-inflammatory agents and antioxidants. The high sugar content in citrus waste also makes it a suitable substrate for bioethanol production, contributing to the development of sustainable biofuel alternatives (*96*). The versatility of products derived from citrus waste goes beyond these traditional applications. Recent studies have explored the use of compounds derived from citrus peel for the development of biodegradable polymers and functional materials to meet the growing demand for environmentally friendly, bio-based solutions in various industries (*83*). The valorization of citrus processing waste has proven to be a promising avenue for the development of a wide range of industrial applications. By utilising the rich chemical profile of these byproducts, researchers and industry players are transforming waste into valuable, high-value commodities, contributing to the transition to a more sustainable and circular economy (*18*).

## CURRENT CHALLENGES AND ETHICAL ASPECTS

The beneficial effects of bioactive compounds in citrus fruits have been explored mainly through *in vitro* and *in vivo* studies, but their therapeutic application is limited by the lack of sufficient clinical evidence. To establish their effectiveness in treating human disorders, further clinical studies on aspects such as intake, metabolism and cytotoxicity of these bioactive chemicals are necessary. The citrus processing industry generates significant amounts of waste, including peels, seeds and pulp residues, which can lead to environmental problems if not treated effectively. The utilisation of citrus waste in various industries, making it a potential resource for value-added products, presents both opportunities and challenges that require careful consideration of ethical and safety aspects.

The use of citrus waste in the food and pharmaceutical industries is subject to various regulations and guidelines to ensure product safety and quality. In the food industry, the incorporation of citrus waste as an ingredient or additive must comply with food safety regulations, such as those set by the Food and Drug Administration or the European Food Safety Authority. These regulatory agencies set guidelines on the acceptable levels of contaminants, the use of food additives and the overall safety of the final product. Similarly, in the pharmaceutical industry, the use of compounds derived from citrus waste must adhere to strict regulations governing the development and production of pharmaceutical and nutraceutical products (*147*).

Ethical considerations also play a decisive role in the utilisation of citrus waste. The potential environmental benefits of diverting waste from landfills or incineration must be weighed against potential negative impacts on local communities or ecosystems. For example, the extraction and purification of certain valuable compounds from citrus waste may require the use of solvents or other chemicals that can have a negative effect on the environment if not handled properly (*148*). Moreover, the safety of the final products derived from citrus waste must be rigorously evaluated. Citrus fruits are known to contain certain compounds such as limonoids and furocoumarins that can have potential toxic effects or interactions with medications. Thorough testing and safety assessments are necessary to ensure that the use of citrus waste in food and pharmaceutical applications does not pose any risks to human health or the environment (*149*).

Consumer acceptance of products derived from citrus waste is hindered by the negative perception of ’waste’. Consumers may be reluctant to buy such products despite their nutritional and functional superiority. To mitigate this problem, it is important to reshape perceptions by emphasising the sustainability and environmental friendliness of these products and highlighting their role in waste reduction and protection of the environment. Another obstacle involves addressing consumer apprehensions regarding the safety and quality of citrus waste-derived products. They may have concerns about contamination or harmful compounds in these products. To address these concerns, rigorous quality control and transparent communication about manufacturing processes and product safety are essential (*22*). Moreover, the effective commercialisation of citrus waste-derived products may require innovative marketing strategies and consumer education about their advantages. By emphasising the nutritional, functional, environmental and economic benefits, manufacturers can change consumer perceptions and improve the acceptance of these innovative products.

## FUTURE PERSPECTIVES

The production of nanomaterials from citrus fruit waste from food processing is a relatively new topic, with limited investigations on the utilisation of citrus peel waste for the production of nanomaterials. Some large micropores in mesoporous cellulose from orange peel were produced by microwave treatment. In another study with citrus waste, nanocellulose with a length and an average diameter of 458 and 10 nm respectively, was obtained. Purified cellulose is obtained from mandarin peel waste, from which cellulose nanofibrils with a width of 2-3 nm were produced. Further literature describes the use of pectin derived from citrus peel waste for the production of microspheres and the use of citrus peel waste as a substrate for the production of bacterial nanocellulose. As a result, the technologies that are most adaptable to the environment will thrive in the future.

Incorporating concepts such as circular bioeconomy and upcycling fruit waste into new resources is essential to improve the sustainability of citrus waste utilization. The researchers investigated different strategies to convert citrus waste into high-value products, like biopolymers, biofuels and dietary supplements. By adopting the principles of circular economy, the importance of closing the loop in the management of citrus waste that reduces environmental impact and promotes resource efficiency is highlighted.

A life cycle was also assessed to analyse the social, environmental and economic aspects of upcycling fruit waste, particularly citrus waste. The aim of this assessment was to evaluate the environmental impact of differnt waste management strategies and highlight the potential advantages of repurposing fruit waste into new resources. They emphasised the importance of considering social acceptability, environmental responsibility and financial viability when implementing upcycling initiatives. Overall, these studies emphasise the importance of incorporating circular bioeconomy principles and upcycling strategies into citrus waste utilisation efforts. By utilising research from online sources, policymakers, researchers and industry stakeholders can develop innovative solutions to address environmental challenges and promote sustainable resource management.

## CONCLUSIONS

Processed citrus waste is a sustainable biowaste that has undergone several valorization studies to recover its energy and matter. The valorization of citrus waste combines eco-innovation, environmental responsibility and financial viability into products such as single-cell proteins, essential oils, activated carbon, packaging films made from citrus waste and food-grade kraft paper, offering significant economic potential by developing new markets. However, challenges remain, particularly in the large-scale implementation of these technologies and the environmental impact of waste disposal if not managed properly. Improper disposal of citrus waste can lead to soil, water and air contamination, with harmful emissions of greenhouse gasses. The present review critically evaluated various valorization techniques, looking at both conventional and non-conventional methods. Although conventional methods are effective, they often involve disadvantages such as long extraction times, heat-induced degradation and the use of toxic solvents. To address these limitations, modern extraction methods such as microwave-assisted extraction, ultrasound-assisted extraction and supercritical fluid extraction have been developed. These techniques are more efficient, require less time and produce higher yields with minimal solvent use. Moving forward, more extensive life cycle analyses and industrial-scale trials are needed to evaluate the long-term sustainability and economic feasibility of these processes. Further research should focus on overcoming the challenges of scalability and optimising biotechnological methods for wider application.
